# Endoscopic Transcecal Appendectomy (ETA): A Literature Review on Risks and Benefits

**DOI:** 10.7759/cureus.40827

**Published:** 2023-06-22

**Authors:** Basil N Nduma, Kelly A Mofor, Jason Tatang, Loica Amougou, Stephen Nkeonye, Princess Chineme, Chukwuyem Ekhator, Solomon Ambe

**Affiliations:** 1 Department of Internal Medicine, Merit Health Wesley, Hattiesburg, USA; 2 Department of Gastroenterology, Texas Tech Paul L. Foster School of Medicine, El Paso, USA; 3 Department of Gastroenterology, Sam Houston State University, Huntsville, USA; 4 Department of Gastroenterology, School of Natural Sciences and Mathematics, University of Texas at Dallas, Richardson, USA; 5 Department of Health and Biomedical Sciences, University of Texas Rio Grande Valley, Edinburg, USA; 6 Department of Gastroenterology, University of Texas at San Antonio, San Antonio, USA; 7 Department of Neuro-Oncology, New York Institute of Technology College of Osteopathic Medicine, Old Westbury, USA; 8 Department of Neurology, Baylor Scott & White Health, McKinney, USA

**Keywords:** appendiceal orifice, efficacy, safety, risks, adverse events, endoscopic submucosal dissection, endoscopic full-thickness resection, endoscopy mucosal resection, endoscopic transcecal appendectomy

## Abstract

Different colorectal lesions have attracted different procedures in their management. One of the novel approaches that have been documented in recent times is endoscopic transcecal appendectomy (ETA). ETA is an endoscopic and less invasive approach to the excision of lesions within the appendix. The appendix is also completely resected in the process. The main aim of this paper is to establish some of the benefits and risks that come with ETA. The study was conducted from a systematic review perspective using the Preferred Reporting Items for Systematic Reviews and Meta-Analyses (PRISMA) protocol, which governs the implementation of systematic reviews. Key considerations in the PRISMA framework used in this article include identifying the articles, screening them, and determining their eligibility and their final inclusion or exclusion based on the specified criteria. To arrive at relevant articles, some keywords were used in the various search engines of the databases that were consulted. Some of the keywords that were used included ETA, endoscopic mucosal resection (EMR), endoscopic full-thickness resection (EFTR), endoscopic submucosal dissection (ESD), adverse events, risks, safety, efficacy, and the appendiceal orifice. It was established that the key benefits of the ETA include the ability to avoid postoperative appendicitis and residual lesions in tissue. On the other hand, some risks that could come with ETA were found to include potential tumor seeding and postoperative bleeding. However, the key study limitation is that most of the referenced studies in this literature review are retrospective case series and case reports that are prone to selection bias. Furthermore, most ETA procedures in this literature review were performed by a few experienced and highly skilled endoscopists, making the ability to make such results generalizable to all endoscopists and patient populations a debatable issue. In the future, there is a need for more multicenter and large studies to be conducted with longer follow-up periods to ascertain the results obtained in this review. This will ensure a more informed decision-making process for or against ETA implementation in real-world clinical environments.

## Introduction and background

With endoscopic instrument advancement continually dominating contemporary healthcare practice, there is an emerging trend in which endoscopic treatment has evolved as the primary option for the treatment of different colorectal lesions. Some of these lesions include submucosal lesions, laterally spreading tumors, and polyps [1]. For such colorectal lesions, the most commonly used procedures include endoscopic submucosal dissection (ESD) and endoscopic mucosal resection (EMR) [2]. However, in situations where lesions involve the appendiceal orifice especially when these lesions invade deep into a patient’s appendiceal lumen, complete resection through ESD or EMR presents significant technical challenges because the distal margins are difficult to fully visualize [3]. These kinds of lesions, therefore, require more invasive surgical approaches.

Recently, endoscopic full-thickness resection (EFTR) has emerged in which EFTR devices are used to create precise incisions in the wall of the gastrointestinal tract, reaching deeper layers and orifices including the full thickness of the tissue. These incisions give endoscopists a better view; thus, they can resect the lesions almost completely. The incisions are then sutured using various techniques [4]. The EFTR procedure is widely accepted, but it comes with mixed outcomes in terms of beneficial effects and associated drawbacks. On the one hand, EFTR is a minimally invasive and single-step procedure. On the other hand, the technique does not promise complete resection in situations where lesions originate deep within an individual’s appendiceal lumen. Hence, the latter limitation makes EFTR associated with cases of postoperative appendicitis [5].

Due to the limitations associated with EFTR, the last demi-decade has witnessed the rise of another minimally invasive procedure that involves total resection of the appendix, endoscopically. This procedure is called endoscopic transcecal appendectomy (ETA). ETA ensures complete resection of the appendiceal lesion regardless of its origin or the extent of invasion of the appendiceal orifice. Given that the appendix is completely resected in ETA, there is no complication of postoperative appendicitis, which could occur in EFTR [6,7]. Thus, the last demi-decade has seen ETA gain significant attention, and ETA is increasingly being used as a treatment for different appendiceal orifice lesions such as chronic appendicitis, appendiceal polyps, appendiceal retention polyps, and colonic sessile serrated lesions [6-8]. Despite the increasing attention on ETA, there is very scant literature available about this procedure. To contribute to and extend the current state-of-the-art in academia regarding this field, the current study aims to conduct a literature review of the ETA technique. The specific objective of this investigation is to uncover the benefits and risks of the procedure by examining studies that focus on its effectiveness, safety, and feasibility or efficacy.

## Review

Materials and methods

This article is a systematic review, and the Preferred Reporting Items for Systematic Reviews and Meta-Analyses (PRISMA) framework was used to guide the selection and analysis of articles used for the review. The databases that were consulted for this systematic review include EMBASE, PubMed, and MEDLINE. The steps taken in the selection process included the use of the following keywords: ETA, EMR, EFTR, ESD, adverse events, risks, safety, efficacy, and appendiceal orifice to search the databases mentioned earlier. The resultant articles from the search were then subjected to a de-duplication process where all the articles that occurred in more than one database were selected out to prevent redundancy. Inclusion criteria were then set, which were used to select articles that were left after the de-duplication process. The inclusion criteria for this study include studies that evaluated the safety and efficacy of ETA intervention in patients with appendiceal lesions. Thus, the focus of the inclusion criteria was the intervention that was ETA and the study population that was patients of any age group who had appendiceal lesions and whose treatment option was ETA. Studies that involved both experimental and control groups were part of the inclusion criteria.

Besides the inclusion criteria, the attribute of outcome variables was considered in this methodology. Here, the studies that qualified to be included in the review were those that had reported parameters such as patient-related benefits of ETA, postoperative appendicitis development (if any), other side effects of ETA, the safety of ETA, and the sustainability of the procedure. When it comes to the research design as an additional consideration in the methodological section, this review relied on both uncontrolled and controlled trials. With regard to data extraction, independent reviewers extracted the information from the selected studies. In situations where the emerging inferences were conflicting, a consensus had to be reached using well-defined parameters. Furthermore, each reviewer was required to center on the outcome variables mentioned earlier to seek and document the information within the scope of the review. Some of the independent variables that had further room for reporting included the gender of the participants, sample sizes, any blinding factors, the duration of the investigation, and the ages of the participants to whom ETA had been performed. The rationale behind this direction was to seek to make inferences on whether or not such factors were likely to contribute to, affect, or alter risks and/or benefits that could be associated with the technique.

**Figure 1:**
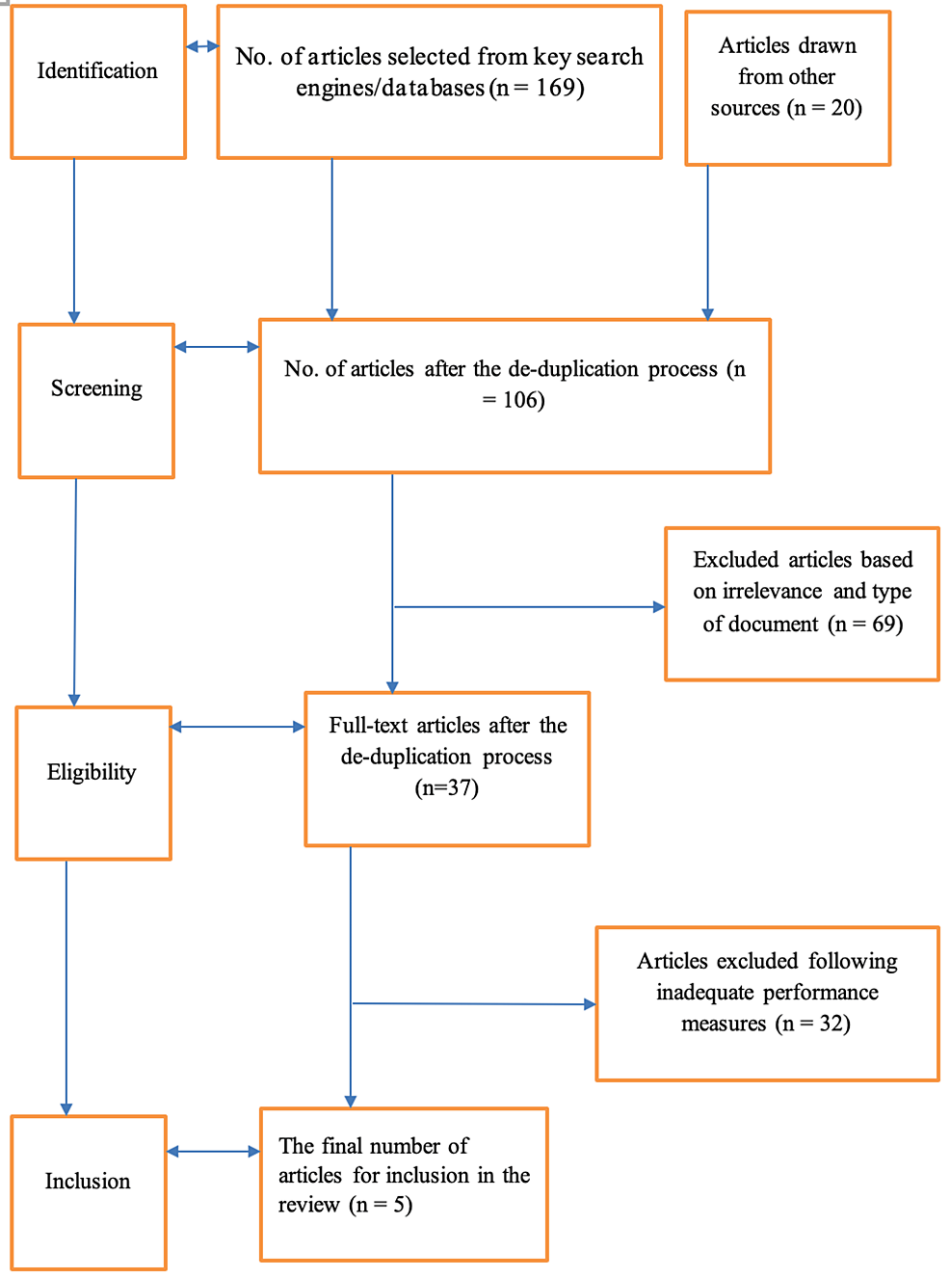
Highlight of the PRISMA framework for article screening and inclusion PRISMA: Preferred Reporting Items for Systematic Reviews and Meta-Analyses

Lastly, the quality of each selected article was considered to inform its final inclusion in the review or otherwise. Thus, on an independent basis, the reviewers would seek to determine any possible presence of bias, including possible allocation concealment, reporting bias, random sequence generation, or performance bias. In turn, any emerging bias risk in articles would be determined as being high, low, or unclear. In case of dissimilarity between the reviewers’ assessment to ascertain article quality, there was further consensus to allow room for more suggestions from a corresponding author, eventually determining whether or not to deem the article as being of high quality, hence its inclusion in the review. The figure below offers a summary of the PRISMA model used in this review in which articles were identified from the databases and screened for eligibility based on the inclusion criteria, and a final decision for their inclusion or exclusion in the study was made. Figure 1 below outlines a PRISMA flow of included studies.

Results

Table 1 below outlines the studies included.

**Table 1:** Studies included in the review ETA: endoscopic transcecal appendectomy

References	Results	Clinical implications
Guo et al. (2022) [6]	In all 13 cases that were the focus, technical success relative to complete resection was documented. With no tumor recurrence and also a period of eight days affirmed to be the median length of hospital stay following ETA implementation, it was concluded that ETA exists as an effective, safe, and feasible procedure for appendiceal orifice lesions’ complete resection.	The study increased the understanding of the need for additional larger multicenter prospective studies aimed at ascertaining the efficacy of the ETA procedure.
Liu et al. (2019) [7]	During and after the ETA procedure, no significant adverse events such as bleeding were reported even after 10 months of follow-up.	ETA exists as a safe procedure, pointing to the need to consider implementing it in future clinical contexts following the identification of eligible candidates.
Muramoto et al. (2021) [8]	Under laparoscopic single-port assistance, ETA was confirmed to be a less invasive procedure compared to conventional laparoscopic resection. Additionally, it was avowed that ETA can be implemented more efficiently and safely compared to situations where endoscopy is implemented alone.	The study formed a baseline and proved foundational in informing the need for future studies to center on the accumulation of additional data relative to the outcomes of the ETA treatment, as well as the importance of considering how to perform ETA safely in eligible patients.
Yuan et al. (2019) [9]	Through a colonoscopic approach, ETA was found to be a promising and successful approach to transcecal appendectomy, with findings suggesting that given that the technique does not come with a skin incision, the option is marked by safety, and so, it is a promising and good alternative for appendiceal disease treatment.	The results were highly contributory to the current state-of-the-art, prompting the need for future clinical studies to examine these beneficial effects with which ETA continues to be associated but focusing on long-term follow-up and larger samples, hence assessing and validating the safety of the intervention.
Atallah et al. (2018) [10]	In this study, ETA was performed successfully, and its feasibility was ascertained relative to its evolution as an alternative technique for addressing and mitigating the problem of the appendiceal orifice.	The findings provided room for future studies to focus on different research populations and also research contexts to inform the sustainability of ETA, as well as shed more light on the technique’s safety when applied to different patient groups in different environments and also with varying degrees of disease severity.

A retrospective analysis was conducted between December 2018 and March 2021 to understand the technical success of ETA, as well as the risk of recurrence, postoperative hospital stay, and postoperative adverse events. With the study population constituting 13 patients, no tumor recurrence was reported in a 17-month follow-up period, with no intra-abdominal abscess, perforation, or postoperative bleeding reported, pointing to ETA’s feasibility, safety, and effectiveness [6]. In a case report involving a 52-year-old female conducted to understand ETA’s safety and feasibility when utilized in treating appendiceal polyps that arise from the inside of the appendix, a 10-month follow-up documented no adverse events, hence confirming ETA’s safety [7] In this study, the procedure itself was performed in two hours 20 minutes. In another case report involving a 72-year-old male who had ETA [8], the total procedure time was 90 minutes, with a follow-up done in two months. In the case report, no adverse events were reported, hence ensuring the safety and effectiveness of the less invasive technique. In yet another case report, ETA was done on a 53-year-old female. The total procedure time was 90 minutes, and there were no adverse events reported, further confirming the safety and effectiveness of ETA [9]. Finally, the effectiveness of employing robotic techniques in performing ETA was investigated [10]. The robotic procedure lasted about 78 minutes alongside laparoscopic assistance. The proof-of-concept purpose was accomplished in the study, demonstrating further the efficacy of flexible robotic systems in accessing anatomies and supporting endoscopic procedures such as ETA, hence the need to consider such conceptual operations in next-generation robotics after preclinical validation [10].

Discussion

An appendectomy is a surgical procedure that involves resection of the appendix and therefore ridding it of any lesions (infections, tumors, inflammation, etc.). The most common indication for an appendectomy is appendicitis, which results from an inflamed appendix. Appendectomy when done for appendicitis is typically an emergency procedure. The appendix is attached to the large intestine, forming a thin pouch sitting in the right and lower sections of the abdomen. In the case of appendicitis, the condition calls for emergent removal of the appendix because a failure to treat the condition could lead to perforation. Historically, appendectomies were done laparotomically. In the last two decades, laparoscopic appendectomies have become the gold standard of care for appendicitis. In the last demi-decade, endoscopic transcecal appendectomies are currently being attempted as an alternative to laparoscopic and laparotomic appendectomies [11].

In this study, the focus was on the implementation of ETA, with particular attention to the literature review of some of the key benefits, indication for the procedure, and the risks associated with the procedure. One of the emerging themes was that decision-making for or against ETA tends to depend on the nature of the condition, particularly its severity. For example, in cases of appendicitis or any appendix lesion associated with appendix perforation and peritonitis, the gold standard of care is laparotomy. In reference to the study’s key objectives, an increase in screening for colon cancer has seen an increase in the number of cases of cecal or appendiceal lesions that involve the appendiceal orifice. Laparotomic and laparoscopic surgical procedures have been the standard intervention for these cases, including partial cecectomy and right hemicolectomy. Despite the promising status of these surgical interventions, however, right hemicolectomy tends to be linked with postoperative complications occurring at a relatively high rate. The implication is that this approach could be deemed excessive for lesions that are relatively benign, including low-grade appendiceal mucinous neoplasms, serrated lesions, and adenomas. On its part, partial cecectomy, when compared to hemicolectomy, can be seen to be less invasive, yet the technique comes with some challenges whereby the surgeon may find it difficult to visualize the margins of the lesions, leading to the performance of right hemicolectomy or extended resection in some instances in a quest to obtain negative margins. It is further notable that converting from laparoscopic surgery to open surgery could be required in some instances, yet such a step comes with a notable increase in surgical trauma and medical costs. As such, unlike surgery, ETA has been documented to constitute certain beneficial effects, especially on the part of patients. For example, in ETA, endoscopists tend to be better placed to visualize the appendiceal orifice lesion’s extent directly. This ability then paves the way for the maximum preservation of the intestine and the orifice lesion.

Another beneficial effect is that in ETA, endoscopists are more likely to exhibit direct access to the appendix and the appendiceal orifice lesion, with the tertiary benefit being the probable facilitation of the appendix’s identification, as well as the reduction of possible injuries that could otherwise pose a risk to other tissues in the surrounding zones, with a particular emphasis on patient populations with a history of abdominal surgery. The third key beneficial feature linked to ETA is that the procedure does not leave scars on the patient’s abdomen, complemented by the position that it comes with no complications often associated with surgical incisions, including wound infection and incisional hernia. In the course of the development of this technique, aspects that are worth noting are that different patients exhibit different needs and preferences, with their disease severities differing from one individual to another. Therefore, while seeking to reap these beneficial outcomes with which ETA continues to be associated, it is key to remember that the decision for or against recommending this procedure needs to occur in consideration of the aforementioned factors that otherwise play a moderating role during the decision-making process. Also, this review established that despite these associated benefits accruing from the implementation of ETA, experimental interventions that have been conducted previously remain scanty. Thus, to ascertain the procedure’s effectiveness and capacity to bring about the abovementioned primary and secondary benefits, especially on the part of both the patient and the provider, more and more scholarly investigations touching on patient populations exhibiting varying demographic features such as age, gender, ethnicity, and sociocultural backgrounds are needed to make more informed conclusions and inferences.

With the simultaneous resection of the appendix and the lesion associated with ETA, the eventuality is that this procedure helps to avoid postoperative appendicitis and also residual lesion in tissue. Here, ETA can be seen to emerge as an alternative that aids in mitigating postoperative adverse events while ensuring complete resection at the same time, leading to direct evidence that the technique is effective, safe, and feasible when it comes to its utilization as a technique for mitigating appendiceal orifice lesions. Also, during the performance of the ETA procedure, with the appendiceal artery managed prudently, the outcome is that the technique leads to the prevention of intra-procedural bleeding. Indeed, in the mesoappendix, the variable nature of the appendiceal artery’s location is a notable point. This variability, particularly, implies that preventing possible appendiceal artery accidental injury requires precise resection of the mesoappendix, with the precaution being more pronounced in patients with a relatively thick mesoappendix. To achieve desirable hemostasis, prior to coagulation, sufficient appendiceal artery exposure is necessary. Another concern is that in the mesoappendix, resecting fat tissue may be difficult especially because of the presence of higher electric resistance in fat. In such a situation, an intrinsic challenge may be vivid if endoscopic intervention is to be adopted because distal looping, if any, could end up hindering the maneuverability of the endoscope [12].

Also, during the performance of ETA, tumor seeding could occur. Therefore, in the course of the entire procedure, the neoplasm is worth keeping intact. To mitigate such a risk of tumor seeding, especially around the lesion, near-circumferential full-thickness resection would be key. Based on these mixed outcomes, the current study established that the performance of the ETA procedure needs to come with great caution, especially in patient populations avowed to exhibit low-grade malignant neoplasms or precancerous lesions in which the appendiceal orifice is involved. In some cases where there is deep infiltration of the appendiceal orifice lesion with clear malignant potential, ETA may also be contraindicated. As such, providers ought to embrace detailed preoperative evaluations to have lesions exhibiting high malignancy excluded. Such evaluations may be in forms such as computed tomography, endoscopic ultrasound, and endoscopy. Besides preoperative assessments, the appendix and the resected lesion are worth evaluating in detail, aimed at governing the patients’ postoperative management following the implementation of the ETA procedure. Furthermore, this review pointed to the need for close follow-up to have potential long-term outcomes of ETA assessed. In the majority of the investigations, however, a key denominator or theme that emerged was that during median follow-ups, ETA does not pose tumor recurrence. Hence, preliminary evidence was arrived at regarding the procedure’s oncological safety when applied to patients diagnosed with appendiceal orifice lesions, but whether these results or observations could also hold if longer-term investigations and follow-ups and larger sample size studies were to be implemented remained unclear, hence the need for such investigations exhibiting long-term follow-up to ascertain these current findings.

Limitations

One of the limitations of this study is that some of the previous scholarly interventions on which it is focused were conducted in the form of retrospective case series investigations. The implication is that whereas the strength of these investigations lies in the fact that they are centered on all cases exposed to the ETA technique in hospitals and also focused on patient populations exhibiting varying lesion pathological types and morphologies and also demographic features, such investigations are prone to selection bias where the authors choose only cases that fit their narrative. Another limitation of the reviewed study is that the majority of the previous scholarly studies consulted had very small sample sizes, suggesting that their ability to be fully evaluated as a novel endoscopic approach remains uncertain. Therefore, in the future, there will be a need for scholarly studies investigating the benefits and risks of the ETA procedure on larger sample sizes to determine whether or not parallels could be drawn between their findings and those established and reported or documented in the current literature. Another aspect is that with the key risk of the ETA procedure involving intra-procedural bleeding, the literature also suggests that the problem could be mitigated through the implementation of endoscopic coagulation. Longer-term follow-up investigations will be key to shed more light on this research outcome. The last limitation of this review is that the majority of ETA procedures investigated within the scholarly articles that were selected or consulted in this literature were performed by a few advanced and highly skilled endoscopists, suggesting that the ability of the findings to be generalized to the rest of the endoscopists remains unclear.

## Conclusions

In summary, the effectiveness, safety, and feasibility of ETA were found to be evident in most studies. The key benefits of the procedure include the probable avoidance of postoperative appendicitis and the avoidance of residual lesions in tissue. However, key risks include tumor seeding and postoperative bleeding. In the future, there is a need for more multicenter and large studies to give more insight into the beneficial effects and drawbacks of the ETA technique as a novel endoscopic approach.
